# Temperament correlates of stigma resistance among patients with mood disorders: a cross-sectional study

**DOI:** 10.3389/fpsyt.2025.1478336

**Published:** 2025-10-27

**Authors:** Rossella Urbani, Anne Chatton, Françoise Jermann, Sophie Favre, Hélène Richard-Lepouriel

**Affiliations:** ^1^ Mood Disorder Unit, Psychiatric Specialties Service, Geneva University Hospital, Geneva, Switzerland; ^2^ Department of Psychiatry, University of Geneva, Geneva, Switzerland

**Keywords:** stigma, stigma resistance, social resistance, mood disorders, temperament

## Abstract

**Background:**

In the realm of mental health, stigma presents a barrier to well-being and social acceptance. However, amidst societal prejudices, stigma resistance emerges as a vital concept, reflecting individuals’ capacity to challenge negative stereotypes and maintain a positive self-concept. This paper explores the dynamics of stigma resistance, its determinants, and its implications for mental health outcomes, focusing specifically on mood disorder patients.

**Methods:**

Adult patients with mood disorders who provided written informed consent were consecutively recruited. Data were collected between 2020 and 2022 at the Mood Disorder Unit of the Geneva University Hospitals. Participants were assessed using the Mini International Neuropsychiatric Interview (MINI), Montgomery–Åsberg Depression Rating Scale (MADRS), Young Mania Rating Scale (YMRS), Internalized Stigma of Mental Illness scale (ISMI), Temperament Evaluation of Memphis, Pisa, Paris and San Diego Autoquestionnaire (TEMPS-A), and the Quality of Life in Bipolar Disorder scale (QolBD). For all scales, higher scores indicate greater symptom severity or higher levels of the measured construct.

**Results:**

In this sample, the majority of patients were women, approximately one-third were single, and about half had completed high school or university education. Most participants were well integrated in the labor market. Multiple linear regression analyses indicated that shorter illness duration, higher hyperthymic temperament scores, and better quality of life were significantly associated with greater stigma resistance. Additionally, a positive trend was observed between internalized stigma and stigma resistance, although this did not reach statistical significance.

**Conclusions:**

Our study highlights the complex interplay of factors influencing stigma resistance among individuals with mood disorders. Understanding these dynamics is crucial for developing targeted interventions to enhance resilience and improve outcomes in this population.

## 1 Introduction

Stigma resistance refers to the intrinsic ability of individuals to deflect or reject stigmatizing beliefs related to mental illness. It involves both avoiding internalization of negative stereotypes and actively challenging them, thus preserving a positive self-concept despite prevailing societal prejudices (1–3). This resistance reflects a dynamic psychological process that empowers individuals to protect their identity and mental well-being in the face of stigma. While self-stigma and its negative effects on outcomes such as self-esteem, social functioning, and treatment adherence have been widely studied, stigma resistance has received comparatively less attention. Firmin et al. (2016) (3) identified positive associations between stigma resistance and hope, quality of life, and recovery outcomes. Additional studies have examined predictors of stigma resistance in individuals with schizophrenia and psychosis, including self-reflection, acceptance of mental illness, and adaptive coping strategies (4–6). Hofer et al. (2019) (7) further demonstrated that resilience is positively associated with stigma resistance and negatively correlated with self-stigma, suggesting that psychological strengths may help buffer against societal prejudice. Stigma resistance is a multifaceted construct influenced by individual, familial, and societal factors. Research indicates that resilience plays a pivotal role in enhancing stigma resistance; for instance, a study on patients with bipolar disorder revealed that higher resilience was associated with lower self-stigma and greater stigma resistance, positioning resilience as a potential target for interventions aimed at reducing stigma (8). Furthermore, internalized stigma can exacerbate the burden on both patients and their families. A study examining adolescents with mental disorders and their families found that internalized stigma significantly contributed to the care burden, highlighting the importance of addressing internalized stigma to alleviate familial strain (9). Interventions targeting stigma resistance have also been explored in medical settings. A systematic review identified various mental health interventions aimed at reducing self-stigma among medical students and doctors, underscoring the need for tailored approaches to mitigate stigma within healthcare professional (10). Additionally, public education initiatives can influence stigma perceptions. A pre-post study assessing the effectiveness of an experiential mental health exhibition found that such interventions could reduce stigma and promote help-seeking attitudes, demonstrating the potential of public education in fostering stigma resistance (11). However, stigma resistance in mood disorders remains insufficiently explored. In a recent meta-analysis, Sum et al. (2024) (12) found that individuals with psychosis reported mild levels of internalized stigma and stigma resistance, and that cultural factor—particularly collectivism—significantly influenced stigma levels. Although the study focused on psychotic disorders, it highlighted the need to consider cultural, economic, and individual factors in stigma research.

An emerging factor in research on BD is affective temperament, especially hyperthymic temperament, characterized by high energy, sociability, and optimism. Often associated with positive functioning, recent findings suggest that hyperthymic traits may contribute to psychological resilience and serve as a protective factor against internalized stigma. For instance, D’Angelo and Steardo (2024) (13) found that internalized stigma was associated with suicidal ideation in BD, with hyperthymic temperament moderating this effect, particularly in sex-specific patterns. In parallel, de Filippis et al. (2022) (14) demonstrated a significant relationship between internalized stigma and dissociative experiences in BD, reinforcing the notion that stigma has complex emotional and cognitive consequences beyond self-esteem, potentially affecting identity cohesion and quality of life. Despite growing interest in resilience in mood disorders, few studies have explored how temperament traits such as hyperthymia might support stigma resistance. The current study addresses this gap by investigating stigma resistance in a clinical sample of euthymic patients with BD and major depressive disorder (MDD). Our aims were to: (1) assess levels of stigma resistance in individuals with mood disorders; (2) identify relevant sociodemographic and clinical correlates; and (3) compare stigma resistance across euthymic BD and MDD groups, with a particular focus on the contribution of affective temperaments—especially hyperthymic traits—to resilience and resistance to self-stigma.

## 2 Methods

### 2.1 Participants

In this cross-sectional study, French-speaking outpatients were recruited in the mood disorder unit of Geneva’s University Hospitals, Switzerland. Inclusion criteria were: (1) a diagnosis of mood disorders, (2) an age of 18 years or above, and (3) fluency in French. Each patient was assessed during three sessions by a psychiatrist and a psychologist specializing in adult mood disorders. Diagnostic was made by a best estimate procedure including a thorough anamnesis (medical histories, family history, onset of the disorder, thymic episodes, and previous treatments) by the psychiatrist and a semi-structured questionnaire (Mini International Neuropsychiatric Interview (MINI), Sheehan et al., 2016) (15) developed to assess the Diagnostic and Statistical Manual of Mental Disorders 5TH edition (DSM-5, APA 2013) (16) criteria, that was completed with a trained psychologist.

### 2.2 Measures

#### 2.2.1 Clinician-assessment scales

##### 2.2.1.1 MINI

The Mini International Neuropsychiatric Interview (15) is a brief structured diagnostic interview for DSM-5 and ICD-11 disorders. The French version demonstrates good interrater reliability (κ = 0.75–0.85) and test-retest reliability (κ = 0.70–0.80) in clinical populations, supporting its use in both research and clinical settings (17).

##### 2.2.1.2 MADRS

The Montgomery-Åsberg Depression Rating Scale (18) is a 10-item clinician-rated scale for depressive symptomatology. Each item is rated on a 0–6 scale, with higher scores indicating greater severity. The French version shows excellent internal consistency (Cronbach’s α = 0.87) and sensitivity to treatment-related changes, validating its use in French-speaking populations (19).

##### 2.2.1.3 YMRS

The Young Mania Rating Scale (20) is an 11-item scale assessing mania severity, with four items rated 0–8 and seven items rated 0–4. The French version demonstrates good interrater reliability (ICC = 0.82) and construct validity with other mania measures (21).

#### 2.2.2 Self-assessment scales

##### 2.2.2.1 ISMI

The Internalized Stigma of Mental Illness Scale (1) assesses self-stigma across five subscales: Alienation, Stereotype Endorsement, Perceived Discrimination, Social Withdrawal, and Stigma Resistance. The French version demonstrates good internal consistency for the total scale (α = 0.87) and subscales (α = 0.68–0.80), with established construct validity in psychiatric populations. In this study, self-stigma refers to the mean score of the four subscales excluding Stigma Resistance, which was analyzed separately as the main outcome variable (22).

##### 2.2.2.2 TEMPS-A

The Temperament Evaluation of Memphis, Pisa, Paris, and San Diego Autoquestionnaire (23) French version Krebs et al., 2006) evaluates five affective temperaments: hyperthymic, dysthymic, cyclothymic, irritable, and anxious. The French version shows high internal consistency (α = 0.70–0.86 across subscales) and good convergent validity with clinical measures of mood disorders, supporting its application in bipolar and depressive populations (24).

##### 2.2.2.3 QoL.BD

The Quality of Life in Bipolar Disorders scale (25) assesses disorder-specific quality of life across twelve domains, including physical health, mood, cognition, social functioning, and identity. The French version demonstrates strong internal consistency (α = 0.89) and construct validity, making it suitable for assessing well-being in French-speaking patients with mood disorders (26).

## 3 Statistical analyses

Descriptive statistics (mean ± SD or percentages) were computed to summarize participants’ characteristics. The primary research question was addressed using multiple linear regression, with stigma resistance as the dependent variable. Candidate predictors included demographic variables (age, gender, civil status, education, employment), clinical characteristics (diagnosis, illness duration, treatment adherence, quality of life), temperament subscales (hyperthymic, depressive, cyclothymic, irritable, anxious), and internalized stigma. Variables correlating with stigma resistance at p ≤ 0.10 or known from prior studies were included; demographic variables were retained as potential confounders regardless of significance. Small category sizes were collapsed to improve statistical power, and categorical variables were dummy-coded. A hierarchical regression was conducted to assess the predictive contribution of blocks of variables: socio-demographic, clinical, and psychological (temperament) variables, with R² change values evaluating block importance. The model’s effect size was calculated using *f*² = *R*²/(1−*R*²). Core regression assumptions—normality, linearity, homoscedasticity, independence of errors, and multicollinearity (VIF ≤ 5)—were checked using standard diagnostic plots (27). For the secondary objective, group differences were assessed with t-tests or Chi-square tests as appropriate. No correction for multiple testing was applied due to the exploratory nature of the study. Of 175 patients, 33 were excluded for missing primary variables. Remaining missing data (up to 18%) were imputed using the Expectation-Maximization (EM) algorithm under a MCAR assumption. Analyses were performed in IBM SPSS (28), with significance set at p < 0.05.

## 4 Results

Descriptive statistics for the total sample are summarized in **Table 1**. Patients were predominantly women (67%), one-third were single, half had a high school or university degree, and an overwhelming percentage (88.7%) were well integrated in the labor market.

**Table 1:** Sociodemographic, clinical and psychological characteristics of patients with mood disorder (N = 142).

Variable	Valeur (format)	Type
Age at assessment	45.4 ± 13.0	M ± SD
Age at onset of illness	29.5 ± 11.6	M ± SD
Duration of illness	16.3 ± 11.5	M ± SD
YMRS total scale	0.8 ± 1.7	M ± SD
MADRS	13.8 ± 9.7	M ± SD
Number of categories of medicines	2.0 ± 1.0	M ± SD
Temperament traits		
-Anxious	1.4 ± 1.1	M ± SD
-Cyclothymic	6.3 ±3.6	M ± SD
-Depressive	3.6 ± 2.5	M ± SD
-Hyperthymic	3.9 ± 2.4	M ± SD
-Irritable	1.8 ± 2.1	M ± SD
Quality of Life (QoL)	35.9 ± 10.4	M ± SD
ISMI subscales		
-Alienation	2.6 ± 0.8	M ± SD
-Endorsement	1.9 ± 0.5	M ± SD
-Discrimination	1.9 ± 0.5	M ± SD
-Withdrawal	2.2 ±0.6	M ± SD
-Resistance	2.6 ±0 .6	M ± SD
Internalized Stigma	2.5 ± 0.5	M ± SD
Gender		
-Male	47(33.0%)	n(%)
-Female	95(67.0%)	n(%)
Education Level		
-Compulsory school	13(9.2%)	n(%)
-High school	20(14.1%)	n(%)
-Apprenticeship	57(40.1%)	n(%)
-University	52(36.6%)	n(%)
Civil Status		
-Single	48(33.8%)	n(%)
-Married or living as a couple	79(55.6%)	n(%)
-Divorced/Widow(er)	15(10.6%)	n(%)
Unemployment		
-No	126(88.7%)	n(%)
-Yes	15(10.6%)	n(%)
Diagnosis		
-Unipolar	85(60.0%)	n(%)
-Bipolar	57(40.0%)	n(%)
YMRS category		
– YMRS ≤ 5	136 (95.8%)	n (%)
– 6 ≤ YMRS ≤ 14	6 (4.2%)	n (%)
– YMRS ≥ 15	0 (0.0%)	n (%)
Hypomanic episode		
– < 7	42 (29.6%)	n (%)
– ≥ 7	3 (2.1%)	n (%)
– Uncertain	12 (8.5%)	n (%)
MADRS category		
– No depression	42 (29.6%)	n (%)
– Mild depression	55 (38.7%)	n (%)
– Moderate depression	42 (29.6%)	n (%)
– Severe depression	3 (2.1%)	n (%)
MDD episodes		
– < 3	47 (33.0%)	n (%)
– ≥ 3	95 (67.0%)	n (%)
Other comorbidities		
– Personality disorder	14 (9.9%)	n (%)
– Anxiety	35 (24.6%)	n (%)
Variable		
– Alcohol and substance use disorder	28 (19.7%)	n (%)
– ADHD	142 (100.0%)	n (%)
– Other (schizo, physio)	2 (1.4%)	n (%)
Current medication/Psychotropic drug		
– Antidepressant	97 (68.3%)	n (%)
– Antipsychotic	56 (39.4%)	n (%)
– Lithium	20 (14.1%)	n (%)
– BZD	52 (36.6%)	n (%)
– Antihypnotic	30 (21.0%)	n (%)
– Other	2 (1.4%)	n (%)
Medication Adherence		
– None	38 (26.8%)	n (%)
– Partial	77 (54.2%)	n (%)
– Full	27 (19.0%)	n (%)
Level of ISMI Alienation		
– Low	66 (46.5%)	n (%)
– High	76 (53.5%)	n (%)
Level of ISMI Endorsement		
– Low	125 (88.0%)	n (%)
– High	17 (12.0%)	n (%)
Level of ISMI Discrimanation		
– Low	118 (83.0%)	n (%)
– High	24 (17.0%)	n (%)
Level of ISMI Withdrawal		
– Low	103 (72.5%)	n (%)
– High	39 (27.5%)	n (%)
Level of ISMI Resistance		
– Low	62 (43.7%)	n (%)
– High	80 (56.3%)	n (%)
Level of internalized Stigma		
– Low	103 (72.5%)	n (%)
– High	39 (27.5%)	n (%)

Continuous variables are presented as mean (M) and standard deviation (SD), and categorical variables as absolute numbers (n) and percentages (%).

No violations of key assumptions for conducting multiple regression were detected. There was no multicollinearity between the predictors, as evidenced by VIF, where no value exceeded 5. In this regard, the maximum VIF value was 2.7. The adjusted R-squared value was 40.7%.

**Table 2** shows the multiple regression results. They show that the duration of mood disorders, the hyperthymic Temperament subscale, and quality of life significantly predict stigma resistance.

**Table 2:** Summary of multiple linear regression analysis for prediction of Stigma resistance in patients with mood disorder.

	Unstandardized β	Std Error	t	Sig	95% CI for β	
					Lower Bound	Upper Bound
(Constant)	3.2	0.4	8.1	<.001	2.4	3.9
Age at assessment	0.002	0.004	0.4	0.7	-0.01	0.01
Gender - Female - Male (Ref cat)	0.1 -	0.09 -	0.6 -	0.5 -	-0.1 -	0.2 -
Civil status - Single, divorced or widow(er) - Married or living together as a couple (Ref cat)	0.02 -	0..08 -	0.2 -	0. 8 -	-0.1 -	0.2 -
Education - Up to high school - University or apprenticeship (Ref. cat)	-0.1 -	0.1 -	-1.3 -	0.2 -	-0.3 -	0.1 -
Diagnostic - Bipolar - Unipolar (Ref. cat)	0.1 -	0.08 -	1.4 -	0.2 -	-0.05 -	0.3 -
Duration of mood disorders	-0.01	0.004	-2.0	0.05	-0.02	0.001
Anxious temperament subscale	0.03	0.04	0.9	0.4	-0.04	0.1
Cyclothymic temperament subscale	0.002	0.02	0.1	0. 9	-0.03	0.03
Depressive temperament subscale	0.03	0.02	1.6	0.1	-0.01	0.07
Hyperthymic temperament subscale	-0.04	0.02	-2.1	0.04	-0.1	-0.002
Irritable temperament subscale	-0.01	0.02	-0.6	0.5	-0.1	0.03
Quality of life (QoL)	-0.03	0.01	-4.4	<.001	-0.04	-0.01
MADRS	-0.003	0.01	-0.6	0.6	-0.01	0.01
YMRS	-0.02	0.02	-0.9	0.3	-0.07	0.03
Treatment compliance - None - Partial - Full (Ref cat)	0.1 0.1 -	0.1 0.1 -	0. 7 1.3 -	0.5 0.2 -	-0.2 -0.1 -	0.3 0.4 -
Internalized Stigma	0.2	0.09	1.8	0.07	-0.01	0.4

Concerning the duration of mood disorders, the negative sign of β indicates a negative association with the dependent variable. Holding all other factors constant, this means that as the duration of the disorder increases, stigma resistance decreases (p=0.046). Similarly, a negative association was observed between hyperthymic temperament, quality of life, and stigma resistance (p=0.04 and p<0.001, respectively). All other things being equal, for one unit increase in these variables, stigma resistance is expected to decrease. The remaining variables did not significantly predict the dependent variable.

The results of the hierarchical multiple regression (output not shown) indicate that the socio-demographic block, entered at step 1, explained 0.1% of the variance in stigma resistance (R square change = 0.001). This contribution, not statistically significant (p=0.995), means that these variables are not necessary in the prediction of the outcome. After entry at step 2 of the clinical block, the R square change value is 0.433. This means that the variables of this block explain an additional 43.3% of the variance in stigma resistance when the effects of the variables of block 1 are controlled for. This is a statistically significant contribution as indicated by the F change value p< 0.001. After entry of the psychological block at step 3, the R square change value is 0.043 or an additional 4.3% of the variance in stigma resistance when the effects of blocks 1 and 2 are controlled for. This contribution is not statistically significant (p=0.08). Given an R-square of 0.477, the effect size was 0.29 which, according to Cohen, is between medium and large. Concerning euthymic unipolar and bipolar patients, their characteristics are described in **Table 3**. Of the 142 patients analyzed, 36 euthymic unipolar (25.3%) and 28 euthymic bipolar (19%) were identified. The two groups were quite similar except concerning drug therapy. Euthymic unipolar received more antidepressants, fewer antipsychotics, and less lithium than euthymic bipolar (p<‘.001, p=0.004 and p= 0.007, respectively). These results were expected.

**Table 3:** Characteristics of euthymic unipolar and bipolar patients [continuous data are summarized as mean M and standard deviation (SD) and categorical data as absolute number n and percentage (%)].

Sociodemographic, clinical and psychological characteristics (n=64)			
Sociodemographics	Euthymic unipolar n= 36	Euthymic bipolar n= 28	P-value
Age at assessment	43.6 (13.2)	44.6 (10.9)	0.8
Gender			0.5
- Male	13 (36.0)	8 (28.6)	
- Female	23 (64.0)	20 (71.4)	
Education level, %			0.4
- Apprenticeship, University	25 (69.4)	22 (78.6)	
- Compulsory school, High school	11 (30.6)	6 (21.4)	
Civil status, %			0.7
- Married or living as a couple	20 (55.6)	17 (60.7)	
- Single, Divorced/widow(er)	16 (44.4)	11 (39.3)	
Unemployment			NA
- No	32 (89.0)	24 (85.7)	
- Yes	4 (11.0)	4 (14.3)	
Clinical and psychological characteristics			
MDD episode			0.4
- Less than 3	14 (39.0)	8 (28.6)	
- Three and more	22 (61.0)	20 (71.4)	
Hypomanic episode - Less than 7	–		–
- Seven and more - Uncertain, unquantifiable or undetermined		20 (71.4) 0 8 (28.6)	
Age at onset of illness	28.2 (11.3)	28.4 (9.6)	0.9
Illness Duration at the time of assessment	16.5 (10.4)	16.5 (10.7)	1.0
Comorbidity: Personality disorder			NA
- No	33 (91.7)	25 (89.3)	
- Yes	3 (8.3)	3 (10.7)	
Comorbidity: Anxiety			0.2
- No	26 (72.2)	24 (85.7)	
- Yes	10 (27.8)	4 (14.3)	
Comorbidity: Alcohol and substance use disorder			NA
- No	30 (83.3)	24 (85.7)	
- Yes	6 (16.7)	4 (14.3)	
Comorbidity: ADHD			–
- No	0 (0)	0 (0)	
- Yes	36 (100)	28 (100)	
Current medication: Antidepressant			<.001
- No	8 (22.2)	20 (71.4)	
- Yes	28 (77.8)	8 (28.6)	
- Current medication: Antipsychotic - No			0.004
- Yes	29 (80.6)	13 (46.4)	
	7 (19.4)	15 (53.6)	
Current medication: Lithium			0.007
- No	33 (91.7)	18 (64.3)	
- Yes	3 (8.3)	10 (35.7)	
Current medication: BZD			0.4
- No	26 (72.2)	23 (82.0)	
- Yes	10 (27.8)	5 (18.0)	
Current medication: Antihypnotic			0.4
- No	28 (77.8)	24 (85.7)	
- Yes	8 (22.2)	4 (14.3)	
Number of categories of medicines	1.7 (0.8)	1.9 (1.0)	
Medication adherence			0.6
- None	10 (27.8)	5 (17.9)	
- Partial - Full	18 (50.0)	17 (60.7)	
	8 (22.2)	6 (21.4)	
Temperament trait			
- Anxious	1.3 (1.2)	1.3 (1.3)	0.8
- Cyclothymic - Depressive	6.1 (3.6)	6.1 (3.9)	1.0
- Hyperthymic - irritable	3.3 (2.4)	2.1 (2.5)	0.07
	4.5 (2.2)	4.2 (2.8)	0.6
	1.9 (2.0)	1.6 (0.7)	0.6
ISMI subscales			
- Alienation	2.3 (0.7)	2.3 (0.7)	0.9
- Endorsement - Discrimination	1.7 (0.5)	1-8 (0.5)	0.4
- Withdrawal - Resistance	1.7 (0.6)	1.9 (0.6)	0.1
	1.9 (0.6) 2.2 (0.5)	2.0 (0.6) 2.4 (0.6)	0.3 0.2
Internalized Stigma	1.9 (0.5)	2.0 (0.5)	0.2
Quality of Life (QoL)	42.5 (8.9)	42.3 (8.9)	0.9
MADRS	5.4 (3.6)	4.7 (3.4)	0.4
YMRS	1.3 (2.2)	0.8 (1.1)	0.3

-: no statistic computed because the variable is a constant.

NA: 2 cells (50.0%) have expected count less than 5.

Quality of Life (QoL) 135.0 (10.3)

## 5 Discussion

The current study aimed to explore the correlates of stigma resistance in patients with mood disorders. Our findings suggest that stigma resistance is higher among individuals with a shorter illness duration, lower hyperthymic temperament scores, and lower quality of life. Interestingly, the association with hyperthymic traits was negative, which contrasts with our initial expectations and calls for a more nuanced understanding of how affective temperaments relate to stigma processes. Neither age nor internalized stigma emerged as significant predictors of stigma resistance, indicating that other psychological or experiential factors may play a more central role in fostering resilience to stigma. Although not statistically significant in the present model, a potential bidirectional relationship between stigma resistance and treatment adherence should also be considered. While greater adherence may foster increased self-awareness and capacity to resist societal biases, the experience of successfully resisting stigma could also empower individuals to remain engaged in their treatment plans. This dynamic interaction highlights the need for interventions that simultaneously support adherence and promote resistance to stigma.

### 5.1 Age and stigma resistance

In our study, age was not a significant predictor of stigma resistance. This means that, in our sample, older participants did not show a stronger ability to resist stigma compared to younger ones. Still, previous research has pointed to the possible role of age in shaping how individuals experience and respond to stigma. For example, O’Connor et al. (6) found that stigma resistance was linked to reduced hopelessness in younger adults (mean age 33), but not in older participants. They also observed a stronger connection between self-stigma and hopelessness among younger individuals. These findings highlight the importance of addressing self-stigma early in the course of mental illness. Future studies could help clarify how stigma resistance may evolve with age, and whether age-tailored interventions might support coping and resilience, especially in younger people who may be more affected by internalized stigma.

### 5.2 Temperament and stigma resistance

The correlation between the hyperthymic temperament subscale and stigma resistance highlights an unexpected and noteworthy finding in our study. Contrary to our initial hypothesis and some assumptions in the literature, individuals with higher hyperthymic traits, typically associated with elevated mood and energy, showed lower levels of stigma resistance. To our knowledge, this is the first documented negative association between hyperthymic temperament and stigma resistance. Simonetti et al. (2023) (29) previously linked high-energy affective temperaments such as hyperthymic, cyclothymic, and irritable with bipolar disorder, and low-energy temperaments with major depressive disorder. Our results suggest that high-energy traits do not necessarily translate into greater resilience against stigma, and may in some cases reflect patterns of engagement or reactivity that make coping with stigma more complex. These findings support the value of integrating temperament assessments into clinical evaluations to better understand stigma dynamics and to tailor interventions accordingly. However, it is important to note that the prevalence of these affective temperaments may differ between individuals with MDD and BD. The bipolar disorder population itself should be further subdivided, particularly distinguishing between BD-I and BD-II patients, as their clinical presentation, symptomatology, and temperament profiles might differ. Furthermore, our study explores the potential association between temperaments, as measured by the TEMPS-A, and the ability to resist stigma. While existing research in this area is relatively limited, theoretical considerations suggest that various temperaments may indeed influence an individual’s capacity to withstand societal prejudices related to mental health. One possible interpretation of this result is that individuals with a hyperthymic temperament may inherently possess qualities such as optimism and high energy levels, which equip them with greater resilience against negative stereotypes and societal prejudices surrounding mental health. Additionally, their naturally elevated mood may contribute to a more positive self-concept, enabling them to confront stigma more effectively. For instance, individuals with a depressive temperament may be more susceptible to internalizing stigma-related beliefs due to negative self-perceptions and low self-esteem. Similarly, individuals with an irritable temperament may experience heightened sensitivity to perceived social rejection, potentially exacerbating stigma-related distress. Likewise, individuals with an anxious temperament may be more prone to anticipating negative evaluations from others and experiencing heightened levels of stigma-related anxiety. Nevertheless, they may be better equipped to resist stigma with effective coping mechanisms and support networks. While theoretical plausibility exists for an association between TEMPS-A-measured temperaments and stigma resistance, empirical research is necessary to investigate this relationship thoroughly. Our findings highlight the hyperthymic temperament as a significant predictor of stigma resistance. The hyperthymic temperament can serve as either a mediator (explaining how stigma influences resistance) or a moderator (buffering the negative effects of stigma). Additional research is needed to determine which role is stronger, but in both scenarios, hyperthymic traits may, in some cases, be associated with a reduced capacity to resist stigma, suggesting a more complex role in stigma-related processes.

### 5.3 Quality of life and stigma resistance

In this study quality of Qol significantly predict stigma resistance. This negative association means that as quality of life increases, stigma resistance decreases. This finding appears counterintuitive, as one might expect that better QoL would empower individuals to resist stigma more effectively, thanks to greater self-confidence, mental well-being, and social support. A higher QoL is often associated with strong social networks and supportive environments, which can provide individuals with the emotional and practical resources to resist stigma effectively (30). On the other hand, in the context of this study, with a sample consisting in a majority of educated and employed women, the negative association between QoL and stigma resistance may be influence by different factors. Educated and socially integrated women may experience less overt stigma due to their social positioning, reducing the necessity for active resistance. In other words, individuals with higher QoL may encounter less direct stigma or feel less compelled to actively resist it, leading to lower stigma resistance scores despite overall well-being.

### 5.4 Self-stigma and stigma resistance

Our study’s unexpected but not significant positive association between internalized stigma and stigma resistance unveils a nuanced and complex interplay between these two constructs. Contrary to conventional wisdom, our findings suggest that individuals who internalize stigma may paradoxically exhibit higher levels of motivation to resist and challenge societal prejudices actively. It implies that internalizing stigma might catalyze empowerment for some individuals, prompting them to confront and counteract stigma more vigorously. This unexpected result challenges existing models and measurement approaches, highlighting the need for a more nuanced understanding of the dynamics between perceived, anticipated, and internalized stigma. Traditionally, internalized stigma has been viewed as a barrier to resilience and well-being, associated with negative outcomes and psychological distress (31). However, our findings suggest a more intricate relationship where internalized stigma may fuel a proactive stance against societal prejudices, contributing to higher levels of stigma resistance. This revelation underscores the complexity of individuals’ responses to stigma and the multifaceted nature of stigma-related processes. It suggests that the experience of internalized stigma may not necessarily be a passive acceptance of societal biases but rather a catalyst for active resistance and empowerment. Thus, our study highlights the importance of considering the intricate dynamics between various dimensions of stigma and resilience, urging researchers to adopt more comprehensive frameworks to capture these complexities effectively. It is possible that internalizing stigma may act as a catalyst for some individuals, motivating them to resist societal prejudices due to psychological reactance or a desire for empowerment. Theories on identity and resilience suggest that rather than passively accepting societal biases, internalized stigma could actually spur individuals to engage in behaviors aimed at confronting and overcoming these stereotypes (32).

### 5.5 Treatment adherence and stigma resistance

Treatment adherence emerged as a significant factor negatively associated with stigma resistance. Specifically, partial adherence was linked to a decrease in stigma resistance, whereas individuals who fully complied with prescribed medications demonstrated higher levels of stigma resistance. Research supports the notion that treatment adherence plays a significant role in stigma resistance among individuals with mental illness. Abdisa et al. (2020) (33) revealed a noteworthy correlation between medication adherence and self-stigma among individuals with mental illness, highlighting that increased self-stigma was linked to non-adherence to medication regimens.

This emphasizes the potential role of treatment adherence in fostering mental health resilience and challenging societal prejudices. Adherence to prescribed treatments may not only enhance individuals’ capacity to resist and counteract societal biases but also contribute to better clinical outcomes and greater insight into their illness. Improved outcomes and increased awareness can further strengthen individuals’ ability to cope with stigma, thereby promoting stigma resistance. The negative association between partial adherence and stigma resistance underscores the need for a comprehensive approach to mental health care that encourages adherence to prescribed medications. This suggests that individuals who consistently adhere to their treatment regimens may be better equipped to resist societal biases, develop deeper insight into their condition, and ultimately experience improved mental health outcomes. Adherence to treatment leads to improved health outcomes, which can enhance a patient’s understanding of their condition. Greater understanding may help individuals resist stigma by promoting self-acceptance and enabling them to educate others. However, this relationship is not clear-cut—factors such as medication side effects, societal stigma, and a lack of support can influence both adherence and understanding. Furthermore, resisting stigma is impacted by external elements like social support and cultural attitudes. While adherence and understanding can aid in resisting stigma, effectively tackling stigma requires systemic changes in mental health awareness and policy. The findings regarding treatment adherence indicate that interventions designed to enhance stigma resistance should prioritize promoting full adherence to prescribed medications (34). Such programs could highlight the significance of medication adherence in building resilience, improving self-awareness, and mitigating the effects of societal prejudice. Moreover, interventions should include coping strategies and offer psychoeducation to strengthen stigma resistance in individuals with mood disorders. By empowering individuals to reject stigma, encouraging peer support, involving families, and addressing practical barriers to treatment adherence, outcomes for individuals with mood disorders can be improved, and stigma-related disengagement from care can be reduced.

### 5.6 Variables without significant associations

Several other variables, including gender, civil status, duration of illness, depressive temperament, and education, did not show statistically significant associations with stigma resistance (6, 35, 36). Our conclusions regarding the absence of significant associations with these variables are consistent with findings from several other studies. Individual differences in coping strategies, social support networks, and resilience may significantly shape an individual’s ability to resist stigma. Additionally, cultural and contextual factors not captured in our study may contribute to the variability in stigma resistance observed across different populations. While our study did not find significant associations with several variables commonly explored in stigma resistance research, these findings underscore the need for a comprehensive understanding of the multifaceted nature of stigma and the diverse factors that may influence individuals’ abilities to resist and counteract societal biases effectively. Cultural and contextual variables, such as the characteristics of the Swiss healthcare system and local norms regarding public stigma, may have influenced the findings of this study. Switzerland’s relatively accessible mental health services and more progressive attitudes toward psychiatric conditions may limit the generalizability of these results to countries with different healthcare infrastructures or stigma norms. Future research should examine how such contextual factors shape stigma resistance across settings.

### 5.7 Directions for future research

The findings of this study comparing the characteristics of euthymic unipolar (MDD) and bipolar patients (BD) align with expectations given the distinct nature of these two mood disorders. Although we did not initially separate MDD and BD groups in the regression analyses, we acknowledge that doing so would have strengthened the validity of our conclusions. Future studies should explore these groups separately in statistical analyses, particularly given their differing symptomatology, prognosis, and response to treatment. This distinction will be essential to better understand stigma resistance within these populations. Additionally, distinguishing between MDD and BD patients in terms of internalized stigma and temperament could clarify how these factors influence stigma resistance differently in each group. The intersection of temperamental traits and mental health disorders presents a complex yet promising avenue for future research and clinical application (37). Understanding how specific temperamental profiles may influence an individual’s susceptibility to societal prejudices and their predisposition to specific mental health conditions could significantly enhance personalized treatment approaches. Moreover, elucidating the relevance of affective temperaments in mood disorder diagnosis, prognosis, and treatment may ultimately lead to more effective interventions and improved patient outcomes. However, it is essential to acknowledge the need for further studies to validate and expand upon these findings. Longitudinal studies exploring the interplay between temperamental traits, societal resilience, and mental health outcomes could provide deeper insights into the mechanisms underlying these relationships. Additionally, investigating the role of affective temperaments in diverse populations and across different cultural contexts would contribute to the generalizability and applicability of these findings in clinical practice. Future studies could explore how specific temperamental traits influence individuals’ responses to stigma-related stressors and their ability to uphold self-esteem and psychological well-being in the face of stigma. In addition to exploring clinical and psychological predictors of stigma resistance, future research should more systematically consider the sociocultural context in which stigma occurs. Our sample, drawn from a French-speaking population at a university hospital in Geneva, represents a linguistic minority in Switzerland, though one that is generally not socioeconomically disadvantaged. This relatively privileged setting may influence both access to care and the ways in which stigma is experienced and resisted. An intersectional perspective is therefore essential: stigma is not shaped by temperament or diagnosis alone, but by the interplay of structural conditions, cultural expectations, and social identities. For example, individuals from migrant backgrounds or French-speaking populations in other Swiss regions may face unique challenges related to economic insecurity, language barriers, or discrimination. Future research should examine how these sociocultural factors interact with individual psychological traits, such as temperament, in shaping stigma resistance. Additionally, sociodemographic variables such as education level and civil status were included in our model based on their potential influence through mechanisms like mental health literacy or social support. While these variables were not significantly associated with stigma resistance in our study, they remain important to investigate in more diverse and representative samples. Further research should continue to explore how sociodemographic and contextual factors contribute to the development of resilience in the face of stigma.

## 6 Limitations

The study provides valuable insights into stigma resistance in individuals with mood disorders but has several limitations. Its cross-sectional design prevents causal conclusions, highlighting the need for longitudinal research to explore how stigma resistance evolves over time. The expected temporal relationship is that high levels of internalized stigma may initially undermine an individual’s ability to resist stigma. Over time, as internalized stigma decreases, whether through personal experiences, social support, or therapeutic engagement, stigma resistance may gradually strengthen. In this sense, lower internalized stigma appears to support a more active stance against stigmatizing attitudes. This dynamic is likely non-linear and influenced by various factors such as life events, individual resilience, and access to supportive environments (1, 38). Additionally, future research should analyze subgroups (e.g., MDD vs. BD-I and BD-II, remission vs. non-remission, inpatient vs. outpatient) for a more nuanced understanding. The sample was predominantly female and employed. A sample dominated by educated women may overstate the impact of education, misrepresent gender effects, and limit the generalizability of the findings. Furthermore, cultural factors were not fully examined, and social acceptability biases may affect reliance on self-reported data. Nevertheless, the effect size value (f2 = 0.29) ranging between medium and large effect is a good estimate of the predictive power of the regression model.

## 7 Conclusion

This study provides valuable insights into the complex relationship between stigma resistance and various factors in individuals with mood disorders. Our findings indicate that shorter illness duration, lower hyperthymic temperament scores, and higher quality of life were significantly associated with lower stigma resistance. Contrary to initial expectations, hyperthymic temperament—often linked to energy, sociability, and optimism—was negatively associated with resistance to stigma. This counterintuitive result invites further investigation into how affective temperament traits may influence the way individuals internalize or respond to societal prejudice. Sociodemographic variables, including age, gender, education level, and employment status, did not significantly predict stigma resistance in our model. Similarly, internalized stigma was not found to be a significant predictor. These findings highlight the complexity of stigma-related processes and suggest that stigma resistance may be more closely related to clinical and psychological factors than to demographic characteristics. The study’s limitations, including the cross-sectional design and the lack of distinction between diagnostic groups (MDD vs. BD), point to the need for future research that considers these variables more thoroughly. Further exploration of the relationship between temperamental traits, stigma resistance, and clinical variables, including symptom severity, comorbid conditions, and treatment setting, will be essential for advancing our understanding of how to best support individuals with mood disorders in coping with stigma. From a clinical perspective, these findings suggest the potential utility of interventions aimed at strengthening stigma resistance, such as psychoeducational programs, peer-support initiatives, and cognitive-behavioral strategies that promote empowerment and self-efficacy. Attention to quality of life and temperamental vulnerabilities may help tailor these interventions to the needs of individual patients. Ultimately, strengthening stigma resistance could play a key role in improving engagement with care and long-term outcomes in mood disorders. Overall, this research opens avenues for future studies to investigate how stigma resistance evolves over time and to develop targeted interventions that address the specific needs of different patient groups.

## Data Availability

The raw data supporting the conclusions of this article will be made available by the authors, without undue reservation.
